# Induced Pluripotent Stem Cells and Their Applications in Amyotrophic Lateral Sclerosis

**DOI:** 10.3390/cells12060971

**Published:** 2023-03-22

**Authors:** Hongmei Du, Zijun Huo, Yanchun Chen, Zhenhan Zhao, Fandi Meng, Xuemei Wang, Shiyue Liu, Haoyun Zhang, Fenghua Zhou, Jinmeng Liu, Lingyun Zhang, Shuanhu Zhou, Yingjun Guan, Xin Wang

**Affiliations:** 1 Department of Histology and Embryology, School of Basic Medical Sciences, Weifang Medical University, Weifang 261053, China; hongmeiduwf@163.com (H.D.); zijunhuo1998@163.com (Z.H.); cyc7907@wfmc.edu.cn (Y.C.); jinqiuhupan@163.com (Z.Z.); 18800460605@163.com (F.M.); 18863662168@163.com (X.W.); 2 Neurologic Disorders and Regenerative Repair Laboratory, Weifang Medical University, Weifang 261053, China; liu08308399@163.com (S.L.); haoyunzh@163.com (H.Z.); zhoufh@wfmc.edu.cn (F.Z.); 18353687185@163.com (J.L.); zly199311@126.com (L.Z.); 3 Department of Pathology, School of Basic Medical Sciences, Weifang Medical University, Weifang 261053, China; 4 Harvard Medical School and Harvard Stem Cell Institute, Harvard University, Boston, MA 02115, USA; shuanhuzhou@gmail.com; 5 Department of Neurosurgery, Brigham and Women’s Hospital, Harvard Medical School, Boston, MA 02115, USA

**Keywords:** amyotrophic lateral sclerosis, induced pluripotent stem cells, disease modeling, drug screening, cell therapy

## Abstract

Amyotrophic lateral sclerosis (ALS) is a progressive neurodegenerative disease that results in the loss of motor function in the central nervous system (CNS) and ultimately death. The mechanisms underlying ALS pathogenesis have not yet been fully elucidated, and ALS cannot be treated effectively. Most studies have applied animal or single-gene intervention cell lines as ALS disease models, but they cannot accurately reflect the pathological characteristics of ALS. Induced pluripotent stem cells (iPSCs) can be reprogrammed from somatic cells, possessing the ability to self-renew and differentiate into a variety of cells. iPSCs can be obtained from ALS patients with different genotypes and phenotypes, and the genetic background of the donor cells remains unchanged during reprogramming. iPSCs can differentiate into neurons and glial cells related to ALS. Therefore, iPSCs provide an excellent method to evaluate the impact of diseases on ALS patients. Moreover, patient-derived iPSCs are obtained from their own somatic cells, avoiding ethical concerns and posing only a low risk of immune rejection. The iPSC technology creates new hope for ALS treatment. Here, we review recent studies on iPSCs and their applications in disease modeling, drug screening and cell therapy in ALS, with a particular focus on the potential for ALS treatment.

## 1. Introduction

Amyotrophic lateral sclerosis (ALS) is a fatal neurodegenerative disease of the central nervous system (CNS) that can lead to progressive degeneration and loss of motor function, and eventually to paralysis. In recent years, it has been found that in addition to traditional motor disorders, its clinical presentation includes behavioral changes, cognitive dysfunction and non-motor symptoms [1,2]. Because of the heterogeneity of its clinical manifestations, location of onset and distribution of motor neurons (MNs), ALS has now been redefined as a systemic disease [3].

The overall crude worldwide ALS prevalence and incidence is between 1.59 and 4.42 per 100,000 person-years [4]. Although the disease is relatively rare, the number of ALS patients is increasing rapidly. ALS mainly includes familial ALS (fALS) and sporadic ALS (sALS) [5]; approximately 10% of cases are fALS and 90% sALS [6], though their clinical presentations are indistinguishable. The cause of most sALS is unknown. The disease appears to occur randomly, without a family history, or even clear environmental risk factors. There is a family history of fALS in patients with the disease. To date, the number of genes known to be involved in fALS has risen to over 40. The most frequent ALS genes are point mutations in superoxide dismutase 1 (*SOD1*) and expanded *GGGGCC* repeats in chromosome nine open reading frame 72 (*C9orf72*). It was first found that *SOD1* caused fALS in 1993 [7]. Mutation of *SOD1* leads to the accumulation of misfolded SOD1 protein, neuronal excitability, mitochondrial dysfunction and oxidative stress, all of which may increase cell death [8,9]. In 2011, it was found that the first intron of *C9orf72* had a large hexanucleotide (*GGGGCC*) repeat expansion on the affected haplotype [10]. Repetitions vary greatly among individuals, ranging from hundreds to thousands. This repetitive amplification of transcription results in abnormal secondary structures in RNA, which gather to form nuclear foci, leading to the interruption of transcription and cytoplasmic transport [11]. Other common genetic causes include mutations of TAR DNA binding protein (*TARDBP*), fused in sarcoma (*FUS*) [12,13]. In addition to gene mutation, other important pathological mechanisms related to ALS include excitotoxicity, mitochondrial dysfunction, axonal degeneration, oxidative stress, neuroinflammation and skeletal muscle degeneration [14,15]. Interestingly, recent studies have reported that gut flora and bioactive metabolites from the gastrointestinal tract that modify CNS diseases can participate in the pathogenesis of ALS [16]. Other studies on ALS patients also support the contention that gut microbiota may be a risk factor [17,18,19]. All these indicate that ALS is not a single disease, but a multifactor one.

Due to the uncertainty of pathogenesis and the diversity of clinical symptoms, early-stage ALS can be difficult to detect. At present, the diagnosis of ALS is based on comprehensive methods, including clinical history, physical examination and confirmatory tests [20]. To facilitate early diagnosis, researchers are developing new biomarkers, such as phosphorylated neurofilament heavy chains (NfHs) and neurofilament light chains (NfLs). Compared with healthy controls, ALS patients show higher concentrations of phosphorylated NfH and NfL in plasma, serum or cerebrospinal fluid (CSF) [21]. Brain and spinal cord imaging, spectral electrophysiological (EEG) topographic maps and magnetoencephalography are being used for diagnosis. However, most of these methods have not yet been clinically validated and remain in the basic research stage.

Generally, patients with ALS die of respiratory dysfunction within 3 to 5 years after diagnosis [22]. ALS cannot be treated effectively. At present, the only US Food and Drug Administration (FDA)-approved drugs are edaravone, riluzole and Relyvrio. However, edaravone can only enhance quality of life, and riluzole only briefly prolongs survival [23,24]. According to a recent study, intravenous administration of edaravone may not achieve further clinical benefits compared to standard treatment alone [25]. Relyvrio can slow down the function decline, but it still needs a longer and more large-scale experiment [26]. A few drugs are being further evaluated, such as Tofersen [27]. Therefore, it is urgent to find new and effective methods to treat ALS.

## 2. Induced Pluripotent Stem Cells

Using four transcription factors (OCT4, SOX2, KLF4 and c-MYC), Takahashi et al. reprogrammed mouse embryonic or adult fibroblasts to form induced pluripotent stem cells (iPSCs) [28,29]. These reprogrammed cells proved to be quite similar to embryonic stem cells (ESCs) in molecular and functional aspects. They are capable of unlimited self-renewal and differentiation into different types of cells. Subsequent studies confirmed that iPSCs could be obtained from ALS patients with genotype and phenotype heterogeneity and had the ability to differentiate into motor neurons [30]. Compared to ESCs, iPSCs might be characterized as having more frequent epigenetic and genetic aberrations as a consequence of the reprogramming process. However, iPSCs come from patients’ own somatic cells, which pose less ethical limitations and a low risk of immune rejection in vivo. This technology is crucial for ALS research. Research into nervous system diseases has long been severely limited because of the invasiveness and high risk of obtaining tissues from the CNS of patients. Some human neurons come from postmortem tissue, which usually represents the final stage of the disease process. However, iPSC technology provides a unique opportunity to obtain human nerve cells, including various neuronal subtypes and glial cells. In addition, this body of research yielded insights into the fALS spectrum and the larger sALS population from ALS patients with genotype and phenotype heterogeneity [31].

We know that iPSCs can be reprogrammed from the three germ cells, such as from fibroblasts (mesoderm-derived), hepatocytes (endoderm-derived) and keratinocytes (ectoderm-derived). Fibroblasts are still the most commonly used somatic cell type for iPSCs generation. However, because skin biopsy remains invasive, patients prefer non-invasive and less invasive procedures, such as those requiring keratinocytes, urine cells and blood cells [32]. These cell types may be more widely used than fibroblasts in the future because they are accessible with ease and non-invasive or less invasive.

Reprogramming strategies for somatic cells have undergone much development. The first generation delivered genes to fibroblasts based on integrated retroviral vectors. Later, people developed strategies to avoid the genetic integration of foreign DNA, including nonintegrated transfer systems using viral or nonviral vectors, e.g., retrovirus, lentivirus, adenovirus, conventional plasmids, recombinant protein, RNA and chemical molecular compounds [29,33,34,35,36,37]. The accelerated development of various programming strategies should ensure the safety and quality of therapeutic applications. Here, we recommend a powerful and efficient system for producing non-transgenic iPSCs under different conditions. It is easy to use and can be applied to various cell types. Sendai virus, an RNA virus, is a safe and effective nonintegrative transfer virus. Without modifying the cellular genome, Sendai-viral human iPSCs can express pluripotency genes, showing demethylation characteristic of reprogrammed cells [38,39]. However, the iPSCs reprogramming technology is still limited by low efficiency. Researchers are actively seeking to improve the efficiency of reprogramming. Hou et al. showed that 7 small molecular compounds could increase the production of iPSCs to 0.2% from mouse somatic cells [40]. Keshi et al. demonstrated that Gadd45a, a heterochromatin relaxer, had a time-dependent function of promoting reprogramming in the early and middle stages of heterochromatin remodeling [41]. Daiki et al. found that the overexpression of the preferentially expressed antigen 12 (Pramef12) in melanoma family members improved the efficiency of iPSC cell derivation [42]. However, because the reprogramming efficiency of iPSCs rarely exceeds 1%, improving the reprogramming efficiency succinctly and efficiently to obtain more iPSCs for downstream applications is still an urgent problem to be solved.

**Figure 1:**
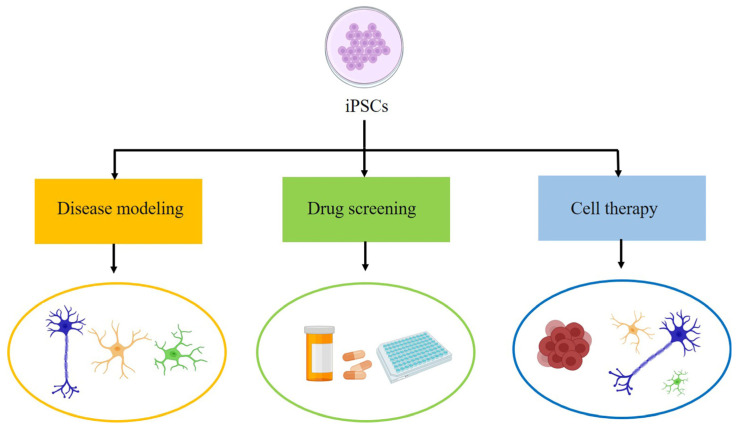
Applications of iPSCs (in the case of ALS). Disease modeling: neurons and glial cells; Drug screening: high-throughput screening; Cell therapy: neural stem cells, neural progenitor cells, neurons and glial cells.

To sum up, iPSCs have broad application prospects in disease modeling, cell therapy and drug screening (Figure 1) because of the ability to self-renew, differentiate into different cell types and retain the genetic background of donor cells.

There are a few reviews on iPSCs and ALS. For example, Lee et al. focus on disease modeling [43]. They discussed modeling ALS and frontotemporal dementia (FTD) with iPSC-derived neurons, described the unique ability of iPSC-derived neurons to capture some of the key features of ALS and FTD and highlight their potential role in drug discovery. Ferraiuolo et al. discussed the different cell types that played a role in the pathogenesis and progression of ALS and described how to use these multiple cell types derived from human iPSCs to identify new therapeutic targets for drug therapy [31]. Lamas et al. described how to effectively generate MNs from human ESCs and iPSCs and discussed the research using iPSC-derived MNs as an ALS drug-screening platform [44]. In this review, we state iPSCs and their applications in disease modeling, drug screening and cell therapy in ALS comprehensively from a larger perspective.

## 3. Modeling of ALS

**Figure 2:**
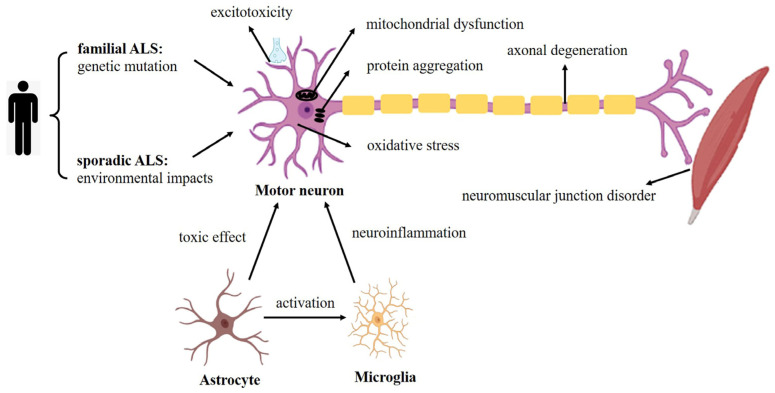
Summary of pathogenesis using iPSC-derived motor neurons and glial cells taken from fALS and sALS patients, which is largely consistent with the pathogenesis of ALS seen in animal models and postmortem tissue.

The advent of iPSCs accelerated ALS modeling in vitro because iPSCs can produce disease-related cell types with the same genetic background of patients with diseases and can summarize the key performance of diseases in in vitro research. It has been demonstrated that multiple cell types are related to the pathogenesis and progression of ALS, such as MNs, astrocytes, oligodendrocytes and microglia. With iPSC technology, MNs and other types of nerve cells derived from ALS patients can be obtained almost unlimitedly. At present, these cell types are successfully generated from somatic cells of sALS and fALS patients, using reprogrammed iPSCs. ALS modeling from human iPSCs (hiPSCs) in vitro is a valuable addition to animal models, and summarizing the effects of these models will help us better understand the pathogenesis of ALS (Figure 2). Here, we focus on modeling ALS using iPSCs-derived MNs, astrocytes and microglia (Table 1).

### 3.1. iPSC-Derived Motor Neurons in ALS Modeling

Traditionally ALS is thought of as related to the progressive loss of motor function, and the most important cells affected during its progression are MNs. iPSCs are becoming a new source of MNs.

Direct differentiation of iPSCs into MNs is the first necessary step toward their subsequent applications. The differentiation protocols for spinal MNs include induction with small molecules, expression of lineage-specific transcription factors and 2-dimensional and 3-dimensional cultures, as well as the implementation of microfluidics devices and co-cultures with other cell types, including skeletal muscle [45]. All of the approaches have advantages and disadvantages. Therefore, the appropriate protocol must be selected according to the research questions. For example, pure and specific subtypes of MNs cultures induced by small molecules or transcription factors expression can be used to study the mechanism of developing MNs and cell-independent disease, and can be easily expanded for high-throughput screening or transplantation. Microfluids and organoids are more suitable for studying the mechanism of non-cellular autonomic pathogenesis and evaluating MNs’ function.

In 2008, it was first reported that iPSCs derived from an ALS patient with a *SOD1* mutation successfully generated MNs and glial cells, which produced a mild disease phenotype [30]. However, do iPSC-derived MNs (iPSCs-MNs) have the same cellular and physiological characteristics seen in vivo? It was shown that the physiological properties of iPSCs-MNs were comparable to those of endogenous spinal motor neurons or ESCs-derived MNs (ESCs-MNs). Assessment of the function of iPSCs-MNs involves evidence of synaptic connectivity, the existence of action potential and continuous action potential trains. iPSCs-MNs displayed morphology, gene expression characteristics and electrophysiological characteristics; formed functional synapses with muscles; and had engraftment ability and sensitivity to de-generative stimuli similar to MNs from other sources [46]. Recently, a new functional evaluation method, high-density microelectrode arrays (HD-MEAs), was employed in research on neuron physiology at different scales, from networks to single neurons to subcellular characteristics. The effects of neuroactive compounds were evaluated by HD-MEAs [47]. The results indicated that iPSCs-MNs had many of the same characteristics as both ESCs-MNs and endogenous spinal MNs. These findings further confirm the possibility of using iPSCs-MNs as the source of MNs for studying motoneuron diseases [48]. Therefore, iPSCs-MNs are being explored for the disease modeling of motor neuron diseases (such as ALS).

SOD1 is an antioxidant enzyme that can protect cells from the influence of reactive oxygen species (ROS) and reduce levels of superoxide [49]. It was found to be a causative gene of familiar ALS, and mutant *SOD1* MNs recapitulated ALS phenotypes. In *SOD1* mutant human iPSCs-MNs, the following features have been observed. One study demonstrated that iPSCs came from patients with *SOD1* gene mutations differentiated into spinal MNs, which showed neurofilament aggregation and neurite degeneration that was closely related to abnormal neurofilament regulation [50]. Another study found that iPSC-derived *SOD1^+/A4V^* MNs exhibited disorders in mitochondria, morphology and movement, and exhibited endoplasmic reticulum stress induction. Further, *SOD1^+/A4V^* hiPSCs-MNs showed shorter cell survival that could be saved through gene correction [51]. Afterwards, the accumulation of misfolded mutant *SOD1* proteins in iPSCs-MNs was confirmed. The proteins played a pathological part in *SOD1* mutant ALS, and the survival rate of ALS MNs decreased, indicating that ALS MNs were more prone to cell death than control MNs or mutation-corrected isogenic ALS MNs [52].

After *SOD1*, *C9orf72* was identified as an ALS-related gene in 2011. European ALS populations are most likely to have this gene mutation [53]. Compared with *SOD1*, *C9orf72* mutation has been studied more in hiPSCs. In 2013, Donnelly et al. confirmed that *C9orf72* ALS iPSCs exhibited toxic RNA foci and repeat-associated non-ATG (RAN) translation pathology; *C9orf72* ALS iPSC neurons were highly susceptible to glutamate-mediated excitotoxicity [54]. Almeida et al. reported the presence of RAN translation in some iPSC-derived human neurons with *C9orf72* mutations. These neurons showed significantly elevated p62 levels and increased sensitivity to cellular stress induced by autophagy inhibitors [55]. It suggested that compromised autophagy function might represent a novel underlying pathogenic mechanism. Later, Westergard et al. found RAN translation in *C9orf72*-ALS/FTD could be driven by neuronal excitation and stress in patient-derived spinal MNs [56]. Dipeptide repeat proteins (DPRs) are generated through RAN translation. They aggregate in neuronal inclusions and might be toxic [57]. Poly GR, one of the DPR, was proved to increase oxidative stress and DNA damage in iPSC-derived MNs [58]. *GGGGCC* repeat expansion compromises nucleocytoplasmic transport in hiPSC-derived neurons with *C9orf72* mutation [11,59]. The expansions are associated with altered endoplasmic reticulum calcium homeostasis and stress granule (SG) formation in iPSCs-derived neurons from patients with ALS and FTD [1]. Impairment of mitochondrial calcium buffering links mutations in *C9orf72* and *TARDBP* in iPSCs-MNs from patients with ALS/FTD [60]. Recent research showed the disruption of endoplasmic reticulum (ER)-mitochondria tethering and signaling in *C9orf72*-associated ALS and FTD. Neurotoxic *C9orf72*-derived DPRs disrupted the VAPB–PTPIP51 interaction to perturb IP3 receptor-mediated delivery of Ca2+ from ER stores to mitochondria, a key ER-mitochondria signaling function. The findings described a new molecular target for *C9orf72*-mediated toxicity [61]. On the whole, mutant *C9orf72* has a variety of pathological ALS phenotypes, including RNA foci formation, DPRs generation, excitotoxicity, SG formation and endoplasmic reticulum mitochondrial destruction. In the iPSCs-MNs of ALS patients carrying *C9orf72* hexanucleotide amplification, researchers studied DNA, RNA, epigenetics and proteins, and then identified novel and known dysfunctional pathways, hoping to find new targeted molecular pathways and target proteins to affect disease progress [62].

Mutations of other genes are also studied using iPSC models. *TARDBP* encodes TDP-43, which is the main protein component of cytoplasmic inclusion bodies found in sALS, and TDP-43 deposition has been proven to have toxic effects on motor neurons. In 2012, the establishment of iPSCs with *TDP-43 M337V* was reported [63]. *TARDBP* hiPSCs-MNs show TDP-43 aggregates in cytoplasm, shorter neurites, mitochondrial calcium buffer junction damage, neuron loss and pathological neurofilament abnormalities, but the neuron loss was not related to TDP-43 mislocation or aggregation [60,64,65]. Among those findings, mitochondrial Ca^2+^ uptake disorder is a common characteristic of ALS caused by *C9orf72* and *TARDBP* mutations. In addition, a study of iPSCs-MNs and *TDP-43* mutant mice determined that cycloguanylic acid (GMP)–AMP synthase (cGAS), a cytoplasmic DNA sensor, caused upregulation of the cytokine profile of neuritis when TDP-43 entered the mitochondria and released DNA through the permeability transition pore [66].

FUS is an RNA/DNA binding protein, which is mainly located in the nucleus and plays a role in many processes, such as transcription, splicing and DNA repair [67]. In ALS-FUS, FUS can undergo liquid–liquid phase separation (LLPS), which is proved suppressed by its nuclear import receptor and arginine methylation [68]. LLPS, followed by cytoplasmic FUS aggregation, is considered to be an important pathogenesis [69]. Liu et al. found significant cytoplasmic mislocation of FUS and more cytoplasmic FUS protein aggregation in iPSCs-MNs produced from the patient with the FUS-P525L mutation compared with the healthy control, which suggested the *FUS-P525L* mutation disrupts FUS protein transport into the nucleus because of a lack of interaction between transportin and the nuclear localization site [70]. Higelin et al. discovered cytoplasmic FUS mislocation, an increased number of FUS positive SGs along neurites and increased DNA damage foci in hiPSC-MNs with *FUS* mutation following hyperosmolar stress or irradiation. The amount of FUS mislocation was positively correlated with the onset of human diseases (the earlier the onset of the disease, the higher the FUS mislocation) and the maturation status of motor neurons [67]. Other studies revealed alterations in autophagy, axonal RNA transport and neuromuscular junction stability in hiPSCs or hiPSCs-MNs carrying a *FUS* mutation [71,72,73]. Recently, it was revealed that in sporadic ALS iPSCs, factors that affect the accumulation of nuclear RNA polymerase II (RNAP II) transcripts regulate the nucleocytoplasmic balance of FUS, and the reduction of RNAP II transcripts can cause the mislocation of FUS to the cytoplasm in ALS patients [74].

However, representing only the pathophysiology of a few patients with ALS (fALS) is a common limitation of these models, which helps explain why drugs that perform well in preclinical studies continue to fail in clinical trials. Therefore, it is urgent to establish a representative model of ALS with less well-understood genetic backgrounds, such as sporadic ALS, since it affects many more patients. Currently the establishment of sporadic patient-specific iPSCs lines is flourishing [75]. Researchers reprogram sALS patients’ fibroblasts into iPSCs, which then differentiate into neurons with a disease phenotype. This provides a cellular model in which to study disease mechanisms and pursue drug discovery. One study demonstrated that MNs derived from 303 sALS patients showed TDP-43 aggregation [76]. Using high-throughput screening, researchers found a small-molecule drug that regulates TDP-43 aggregation and proved that patient-derived iPSCs could be used to screen drugs based on disease models. Another study revealed that, across the broad gene spectrum of differentiated MNs in patients with sALS, mitochondria participated in the establishment of autonomous mechanisms related to sALS [77]. Sun X et al. also demonstrated that TDP-43 protein change, neurofilament inclusion and mitochondrial distribution damage were common early pathologies in sporadic ALS [78]. Researchers also developed a smart phone system to conduct a multiomic analysis of the spinal cord neurons differentiated from iPSCs of fALS and sALS patients, including whole genome sequencing, RNA transcriptomics and proteomics, generating comprehensive data for analysis in hopes of establishing a new model to better understand the mechanism of disease [79].

Based on the above findings, we explored the relationship between the genotype and phenotype of ALS disease and discovered that nucleocytoplasmic transport defects were detected in hiPSCs-MNs of *C9orf72* and *TDP43* mutant patients, but not in *SOD1* ones, indicating that different genotypes may cause MNs degeneration through different mechanisms. One interesting study indicated that hiPSCs-MNs derived from some *SOD1*, *FUS* and *C9orf72* all exhibited hyperexcitability associated with decreased cell survival, despite differences in firing patterns [80], which suggested that different genotypes may express similar phenotypes. These lines of evidence demonstrate the overlap and heterogeneity of pathological phenotypes in iPSC models of ALS. It is worth noting that iPSCs and their derivatives may retain some epigenetic memory of donor cells under the influence of the cells of origin, but whether this will affect the pathological expression of iPSC-differentiated cells, resulting in some negative functional effects, still requires further study and analysis.

In conclusion, the development of ALS modeling with patient-iPSCs provides an exciting opportunity to probe molecular phenotypes underlying disease pathology and mechanisms of ALS within human cells. What is more, it provides a new broader scope for drug screening. This technology can model not only familial, but also sporadic ALS, expanding the scope of study and offering personalized or targeted treatment methods. These have greatly broadened the research vision of ALS.

### 3.2. iPSC-Derived Glial Cells in ALS Modeling

In 1996, glutamate transport defects were found in the spinal cord and motor cortex of ALS patients. This defect seems to be due to the selective deletion of astrocyte-specific glutamate transporter EAAT2 [81], which confirms that astrocytes may be associated with ALS, and this non-cellular autonomous contribution is important for pathogenesis. Subsequently, several studies demonstrated that ALS disease progression is regulated by neurons and other non-neuronal cell subtypes; that is to say, the effect of ALS is not limited to neurons. We will learn more about the onset, progression, and symptoms of ALS by studying the impact on glial cells, which should help us better understand the complex relationship between different nerve cells in healthy people and patients with ALS. However, here we focus on the progress of hiPSCs-derived glial cells in ALS patients. hiPSCs-derived astrocytes and microglia provide a promising platform from which to investigate the effects of ALS mutation on glial cells and the interaction between glial cells and MNs.

The method of differentiating astrocytes from hiPSCs usually consists of four main phases, as described by Tyzack et al. [82]. Unlike astrocytes derived from hiPSCs, which have been studied for more than 10 years, microglia derived from hiPSCs have not been studied until recently. Typically, microglial protocols for differentiation from iPSCs also consist of four steps, as described by Muffat J et al. [83]. Human iPSCs-glial cells are similar in terms of morphology, gene expression and function to human primary glia.

Astrocytes are the most abundant type of glial cells in CNS and play roles in the development and function of neurons. Their dysfunction is related to several nervous system diseases (such as ALS). In 2017, researchers observed a greater accumulation of p62 in MNs treated with patient astrocyte-conditioned medium, along with increased expression of SOD1. These results suggested that patient astrocytes might regulate the death of MNs by impairing autophagy mechanisms [84]. In 2019, Anastasya et al. showed that human astrocytes with mutations of *C9orf72* or *SOD1* reduced the survival of MNs. Dysfunction and neurotoxic factors released by astrocytes may cause their toxicity [85]. One study showed that mutated astrocytes show RNA foci and dipeptide duplication in ALS pathology, which was consistent with the pathology of *C9orf73* mutant ALS. In addition, during co-culture, the output of action potentials by MNs was damaged, which could be reversed by the repeated amplification of excision *C9orf73* mediated by CRISPR-Cas-9 [86]. iPSCs-MNs and astrocytes were studied in Cu/Zn-SOD1^L39R^-linked ALS patients. In MNs cultured with astrocyte-conditioned medium, the expression of the SG marker protein G3BP1 was increased, the expression of caspase 3/7 was upregulated, and autophagy was overactivated. The secretome of astrocytes from patient iPSCs reflected significant oxidative stress in MNs [87]. In 2022, it was proved that the cytokine secretion by a sALS patient’s iPSCs-derived astrocytes was regulated by the mTOR/ULK1/Beclin-1 pathway. Exogenous inhibition of the mTOR–autophagy pathway increased the levels of cytokine, which reduced the viability of motor neurons [88]. These findings prove that iPSCs-derived astrocytes can express dysfunction of themselves and modulate MN death in a non-cell autonomous manner, such as via the autophagy pathway.

Microglia are immune cells of the CNS. They play a role in various aspects of brain development, plasticity and stability through specific recognition, phagocytosis and degradation. A recent report indicates that microglia affect neurofilament deposition in ALS iPSCs-MNs. Using iPSCs derived from a group of identical twins with ALS, astrocytes and microglia were evaluated for their effects on neurofilament protein expression and accumulation in MNs. It was found that the transcriptional level of three neurofilament subtypes of MNs increased. It was further seen that astrocyte-conditioned medium and microglial-conditioned medium influenced the neurofilament deposition of MNs. These results suggest that neurofilament pathology might be modified by glial-secreted factors in ALS iPSCs-MNs [89]. Another report indicated that the *P525L* mutation caused FUS protein to be mislocated from the nucleus to the cytoplasm. Homozygous *P525L* mutation disrupted the transcriptome, and many differentially expressed genes were related to the function of microglia [90]. Whole genome sequencing showed that interleukin 18 receptor helper protein 3’UTR (IL18RAP 3’UTR) variation was greatly enriched in the non-ALS genome and was linked with a fivefold reduction in the risk of ALS. Further studies demonstrated that IL18RAP 3’UTR variants might prevent ALS, because they can reduce the neurotoxicity of microglia derived from human iPSCs [91]. The above-referenced studies confirm that microglia are involved in ALS pathophysiology.

Oligodendrocytes wrap the axons of neurons to form a thick myelin sheath, which enables quick transmission of electrical signals. Oligodendrocytes communicate with other cell types of the CNS in a variety of ways, including by secreting nutrient factors to regulate the size of neuronal axons and the distribution of ion channels in axons [92]. Although oligodendrocytes can be produced from hiPSCs, studies focus mainly on demyelinating diseases, such as multiple sclerosis. Because that process is reported rarely in ALS, we will not go into detail here.

Besides acting on neurons, glial cells also interact with each other. Activated microglia secrete Il-1 α, TNF and C1q to induce type A1 astrocytes (a subtype of reactive astrocytes), causing them to lose the capacity to improve neuronal survival, growth and synaptogenesis, finally leading to the death of neurons [93]. Gaining a deeper understanding of the molecular interactions between cells in ALS will lay a biological foundation for determining correct drug targets to successfully attack ALS diseases. Studying intercellular communications will better arm us to combat ALS.

The study of hiPSCs-derived glial cells has improved our comprehension of the pathology and mechanisms of ALS. ALS affects the entire neural network as a multi-factorial disease, including MNs and glial cells. Although interactions among glial cells have been demonstrated in ALS, it is a puzzling problem what induces neuroinflammation and how these changes lead to neurodegeneration. We suggest that ALS treatment strategies must target MNs and glial cells to eliminate nervous system damage. It will be essential to systematically investigate glia across different genotypes. The control of neuroinflammation by regulating the communication between MNs and glial cells is currently an active area of study regarding therapeutic strategies for ALS.

In summary, the modeling of ALS using iPSCs and research into changes in disease-associated cells not only lay a sound theoretical basis for pathogenesis, but also facilitate drug screening and cellular treatment of ALS. Continuing to expand the collection of phenotypes of related cell types from iPSCs in ALS patients, tracking the progress of the disease and summarizing physiological and pathological manifestations will all help achieve valuable insights into the physiological and pathological characteristics of ALS, and open up prospects for identifying new mechanisms and provide new perspectives for determining new treatment methods.

## 4. Cell Therapy of ALS

Given its low endogenous repair ability, degeneration and damage of the CNS are difficult to cure. iPSCs have unlimited self-renewal ability and can differentiate into ectodermal cells, providing a feasible source of nerve cells for transplantation, and the transplantation of neural derivatives of iPSCs is a promising approach for clinical applications of cell therapy for ALS.

Because MN loss is one of the main features of ALS, replacing damaged MNs is a tempting strategy. However, cultured MNs are generated mainly for modeling ALS diseases; few studies have directly transplanted cultured MNs to replace damaged MNs. The reasons may include that transplanted MNs no longer divide and must form neuromuscular synapses by precise long-distance axon pathfinding to the target muscle and become involved in the formation of physiologically functional synapses. Thus, direct replacement of degenerating MNs with iPSCs-MNs may not be a feasible treatment strategy for ALS.

There is mounting evidence that the curative effects of cell therapy are based on paracrine effects, rather than the survival of transplanted cells [94]. Causes of MN degeneration in ALS include defective neuron–glial communication or lack of nutritional support [95,96]. Transplantation of iPSC-derived glial cells to improve the environment of MNs may be an effective treatment. Sareen et al. differentiated iPSCs into neural progenitor cells (NPCs) with a spinal cord phenotype. Transplanted iPSC-derived NPCs survive well in the spinal cord and differentiate into astrocytes [97]. Takayuki et al. cultured iPSCs into glial-rich NPCs and transplanted them into an ALS mouse model. The transplanted cells differentiated into astrocytes, which upregulated the survival of neurotrophic factors and activated cells and prolonged the life of mice in the treatment group. The results demonstrate the efficacy of iPSC-derived glial cells in the treatment of ALS [98].

iPSCs can differentiate into NPCs or neural stem cells (NSCs) under different transformation conditions. Then, NPCs or NSCs can differentiate into neurons, astrocytes or microglia. In theory, transplanted iPSC-derived NPCs (iPSCs-NPCs) or iPSC-derived NSCs (iPSCs-NSCs) could directly replace dead MNs in the host and reestablish interconnections to resume motor control of voluntary muscles in ALS patients. Transplanting human iPSCs-NPCs or iPSCs-NSCs into the brain or spinal cord to take the place of lost cells, regulating the injurious environment to protect and regenerate host neurons, is a potentially significant therapeutic strategy for ALS. In 2013, it was proved that NPCs derived from human iPSCs survived and differentiated upon transplantation into a rat model of ALS [99]. Monica et al. then isolated a group of specific NSCs from human iPSCs and evaluated the effect of the NSCs on ALS mice after intrathecal or intravenous injection. The results showed that neuromuscular function was improved, and the motor unit pathology of the ALS mice in the treatment group was ameliorated, and survival was significantly prolonged compared with the control ALS mice [100]. In 2016, it was found that the transplantation of iPSCs-NSCs fragments into SOD1G93A transgenic ALS mice protected MNs, promoted their ability to maintain the integrity of neuromuscular junctions, induced new axon germination and reduced the proliferation of glia cells. Co-cultured toxic ALS astrocytes with human ALS-derived MNs, iPSCs-NSCs can enhance neuronal survival and the axon growth of MNs and act on autonomic and involuntary ALS disease characteristics [101]. Recently, Rosati et al. actively sought the establishment of stable human iPSCs-NSCs lines for cell therapies [102]. Nizzardo et al. explored the mechanism by studying the role of the intraspinal injection of iPSCs-NPCs in SOD1G93A transgenic rats. The results demonstrated that the transplantation of iPSCs-NPCs clearly preserved MNs, slowed disease development and prolonged survival in all treated animals. Application of iPSCs-NPCs protected the perineural net surrounding the preserved MNs [103].

In summary, which type of cell product (single cells, aggregates or organized tissues) is applied to cell replacement therapy will vary according to the situation of the disease. For instance, purified single-cell suspensions of dopamine neurons are most suitable for use in Parkinson’s disease. However, aggregates of NSCs/NPCs may be more available for transplantation in ALS. Studies have shown that iPSCs-NSCs and iPSCs-NPCs can effectively bind to host tissue parenchyma after transplantation, differentiate into glial cells and neurons, and exert positive effects through the release of growth factors and immune regulation [104]. However, there are still a number of barriers to overcome, including the duration of NSC and NPC survival within tissues and the potential for tumorigenicity.

**Figure 3:**
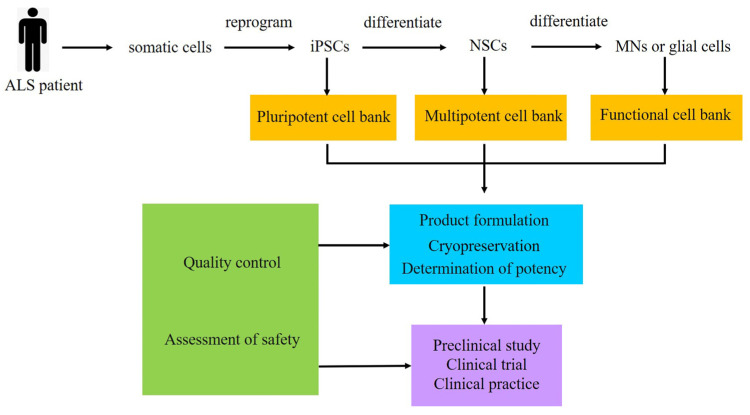
Ideas for cell therapy of ALS with iPSCs. iPSCs: induced pluripotent stem cells, NSCs: neural stem cells, MNs: motor neurons.

However, how to transfer cell therapy from laboratory and experimental animals to clinical application is still a difficult problem that requires considerable efforts. In order to obtain accurate and repeatable data in future preclinical and clinical trials, it is necessary to establish a standardized cell preparation and transplantation scheme (Figure 3) [105,106]. Although autologous iPSC therapy has potential benefits, it also has some limitations, such as higher medical costs and a long-time preparation. So, it is necessary to establish cell banks. When preparing cells, we need to take into account the differences between cell lines, which usually include genetic variation and epigenetic differences. The genetic variation is related to the genetic background of the donors and the mutations in the process of cell derivation, while the epigenetic difference is related to the epigenetic memory of the cells, culture conditions and reprogramming methods [107]. The iPSC lines have shown greater diversity than ESCs. One way to solve the variability of donors is to significantly increase the number of copies in the control group and patients. The Answer ALS (AALS) analyzed the variability of hiPSC donors by analyzing data from MNs differentiated from more than 1000 patients and control groups to obtained cell lines with different characteristics [79]. This is conducive to subtype identification and a better understanding of the underlying mechanism of disease. The impact of potential abnormalities obtained by hiPSCs should also be considered. Chromosome aberration is generally considered to be a serious hazard, so hiPSCs carrying this change are usually excluded from clinical use. However, there is no routine test for epigenetic aberration [108]. In addition, advances in genome engineering have significantly improved the ability to control the genetic variability between individuals by correcting disease-causing mutations in hiPSCs to generate isogenic controls [109]. In order to reduce immune rejection after transplantation, according to the experience of bone marrow transplantation, it is feasible to match the types of three major HLA loci, including A, B and DR, between the recipient and donor [110]. At present, the most realistic hiPSC treatment method is based on collecting hiPSC stock from various HLA homozygous donors. Some HLA homozygous hiPSC bank projects have been started in Japan, Europe and the United States.

Some iPSC-based cell therapies are entering clinical trials, but these therapies are still in their early stages, and safety and effectiveness remain critical issues. In order to carry out this research in a safe and responsible manner, it is crucial to understand the developmental mechanisms underlying cell differentiation, multicellular cell interaction, tissue homeostasis and repair in each case. In conclusion, continuous clinical trials, a great quantity of patients and long-term follow-up should be properly adjusted to obtain significant insights into the efficacy, safety and feasibility of stem cell therapy for ALS in the future. Much work must be undertaken to achieve the maximum benefit of stem cell therapy for ALS.

## 5. Drug Screening of ALS

It has been 15 years since the advent of iPSCs technology, and its applications continue to expand. Given the progress of cell therapies using iPSC derivatives and the use of disease-specific iPSCs for pathological analysis, the safety and efficacy of drug development using iPSCs is booming. Using iPSCs from patients’ somatic cells, researchers can directly obtain pathogenic cell types with the same disease mechanisms from patients for drug development. Several groups have undertaken human iPSC-based drug development and drug repositioning for ALS (Table 2), which has the potential to overcome the species-related restrictions of animal models.

Many current ALS drug-screening platforms were built on iPSC technology. Imamura K et al. generated iPSCs-MNs from ALS patients with *SOD1* mutations. Using this phenotypic assay, they performed a high-throughput screen of 1416 compounds. It was found that Src/c-Abl inhibitors ameliorated the degeneration of MNs in ALS. Src and c-Abl are ubiquitous nonreceptor tyrosine kinases that directly block targets of Src/c-Abl, like bosutinib. Results showed that the positive effect of bosutinib might be related to an increase of autophagy, and bosutinib treatment may reduce the amount of misfolded SOD1 protein in MNs. Among iPSCs-MNs from 11 patients with fALS or sALS, bosutinib treatment increased ALS MNs survival in all patients with fALS and in some with sALS [52]. Bosutinib has been administered to ALS patients in clinical trials to assess the safety and tolerance [116,117].

Fujimori et al. developed a case clustering system capable of subdivision according to the in vitro characteristics of heterogeneous sALS models. They evaluated multiple-phenotype rescue using drug screening and considered ropinirole (ROPI) a candidate [118]. ROPI is a non-ergoline dopamine, which was identified from 1232 FDA-approved drugs. ROPI inhibits the ALS-related phenotypes FUS/TDP43 mislocation, stress particle formation, MN death/injury and neurite regression in iPSCs-MNs from ALS patients, especially in sALS. ROPI had a positive effect on the inhibition of neurite retraction and cell death. However, ROPI does not appear to have a sufficient effect on fALS patients with the *SOD1* mutation. The mechanism of action of ROPI could be (1) to inhibit oxidative stress, (2) to inhibit TDP-43 and FUS aggregation or (3) to improve mitochondrial function [111]. Considering the results of better brain delivery and tolerability of ropinirole hydrochloride, including possible adverse effects, ropinirole hydrochloride was finally found. Now, ROPI hydrochloride has been used in ALS clinical trials [119].

Brian et al. found that the hyperexcitability detected in ALS patients was reproduced in iPSC-MNs with *SOD1*, *C9orf72* and *FUS* mutations, using multielectrode arrays and patch-clamp recordings. When tested in *SOD1*-mutated ALS cases, the Kv7 channel activator not only blocked hyperexcitability, but also improved motor neuron survival in vitro [80]. Retigabine (ezogabine) was confirmed as a prospective candidate for ALS therapy; it is a potassium Kv7 channel activator approved by FDA for epilepsy. Boudewijn et al. used computational models to explain complicated excitability changes in a recent study of 18 ALS patients and analyzed how retigabine worked for human myelinated motor axons. Compared to baseline, the difference in excitability after administration was well simulated by the hyperpolarization shift of the half-activated potential of the slow potassium (K^+^)-channel. These observations confirmed that ritigabine slows K^+^-channel gating [120]. Recently, a randomized clinical trial demonstrated that ezogabine reduced the excitability of cortical and spinal MNs in ALS participants [121]. Further research is needed to ascertain whether long-term therapy slows disease progress and continues to reduce excitability.

In addition to the above three drugs, iPSC-derived cells also provide tools for the study of other drugs or small molecular compounds. Experiments using iPSCs technology to extract compounds for pathological analysis are focused on the treatment of ALS. Tsuburaya et al. screened about 160,000 compounds and identified some low-molecular-weight compounds that inhibit the interaction of 122 SOD1mut and SOD1-Derlin-1, greatly improving the pathology of patient-induced multipotent stem-cell-derived MN and ALS in model mice. This indicated that the SOD1-Derlin-1 interaction contributes to the pathogenesis of ALS and is a prospective drug target for ALS [112]. Fang et al. performed a high-content screen to identify molecules that changed SG properties in hiPSCs-MNs. They found that several hit compounds containing extended planararomatic moieties could prevent the recruitment of TDP-43 and FUS into SGs, and proposed that compounds with planar moieties contain a target point for small-molecule therapy for ALS/FTD [113]. Kuta et al. demonstrated that histone deacetylase (HDAC) inhibitors, such as SAHA, RGFP109 and arimoclomol, reduce the loss of the disease marker nucleus FUS, and in iPSCs-MNs with the *FUS^P525L^* mutation, HDAC inhibition restored the DNA repair reaction, pointing out a variety of neuroprotective mechanisms of HDAC inhibitors and arimoclomol [114]. Choi et al. discovered that inhibition of the PP1-Drp1 cascade could prevent ALS-related symptoms, including mitochondrial breakage, mitochondrial complex I injury, axonal degeneration and cell death. These findings suggest that the regulation of PP1-Drp1 activity might be a potential target for ALS treatment [122]. Kato et al. demonstrated that STAT3 inhibitors (such as niclosamide) can prevent TDP-43 mislocation and degradation, and reduce morphological changes, which suggested niclosamide as a therapeutic candidate for ALS [115].

There is still the need to clarify whether the mechanisms of action of these drugs and compounds share a common pathway and how much the responder populations overlap for each drug. To this end, it is crucial to subdivide ALS according to clinical features, biomarkers and epigenomic information and to confirm the most appropriate drugs for each subcategory.

In summary, because discovery of new drugs is time-consuming and costly, and the success rate very low, while traditional transgenic animal models and immortalized human cell lines cannot offer an accurate system to comprehensively evaluate the exact impact of drugs on ALS, the emergence of iPSCs technology provides a new modality with which to address these problems. iPSC modeling provides a platform for the screening of ALS-related phenotypes, which will also accelerate ALS drug screening and translation to clinical trials. Precision medicine approaches using iPSCs have already been applied to ALS, and further development of iPSCs as validation tools for disease modeling and drug discovery will require prediction of patient efficacy from phenotype-saving drugs in iPSC-derived models to allow stratification of patients on the basis of drug response rather than clinical characteristics.

## 6. Other Applications

As technologies continue to develop, iPSC applications are entering a new phase. Combined with iPSC-based models, a considerable number of new strategies have been expanded to accelerate disease modeling, cell transplantation and drug screening in ALS, including three-dimensional (3D) culture technology, microfluidic technology, single-cell RNA sequencing, genome editing and artificial intelligence (AI).

In addition to single-cell culture and differentiation, multicellular and 3D culture compositions are now also used to study the interactions of disease-related cells. The 3D neuromuscular junction (NMJ) organ-chip model was used to co-culture the musculoskeletal tract and the hiPSCs-MNs of patients with sALS, which verified that bosutinib combined with rapamycin could reduce muscle contraction in sALS cultures. The ability to combine microfluidics and iPSCs technology opens up a new path to study molecular and cellular ALS phenotypes in vitro. Microfluidic devices can simulate NMJ and provide different spaces for different cell types, enhancing control of the cellular microenvironment. In addition, they can be used with 3D cell culture, which improves the function and maturity of NMJ [123]. Namboori et al. used single-cell transcriptomics to reveal dysfunctional pathways and respective isogenic controls in degenerating MNs from ALS patient iPSCs. They discovered that activation of SMAD2 (a downstream mediator of TGF-β signaling) was an important driver of degeneration in *SOD1* iPSCs-MNs and demonstrated the utility of single-cell transcriptomes to map disease-related gene regulatory networks [124]. CRISPR genome editing offers an advanced technology capable of correcting or introducing any intended mutation and can also knock out or remove genes according to the needs of functional research, when combined with iPSCs technology. Eric et al. proposed a method combining CRISPR-Cas9 editing and droplet digital PCR (ddPCR) technology, coupled with a locked nucleic acid (LNA) probe. Using this method, they knocked-in or corrected the known genetic variation of *SOD1* and *FUS* in hiPSCs, proving that the edited cells could effectively differentiate into corresponding MNs. This appears to offer great potential to develop human ALS models in culture dishes [125]. To assist diagnosis, Keiko et al. built an AI-based ALS prediction model using iPSCs. Deep convolutional neural networks were used to analyze spinal cord motor neuron images of iPSCs from healthy controls and patients with ALS, and an algorithm was built and confirmed to distinguish them. The prediction model of the deep learning algorithm based on iPSCs technology can support the diagnosis of ALS and provide prospective treatments meriting future research [126].

In summary, advances in technology continue to promote the research and development of ALS diagnosis and treatment. In the future, automated systems will be used for cell culture and high-throughput screening of candidate compounds for large-scale preparation, and deep learning techniques may be helpful in combining phenotypic imaging and computation with microarray methods for large-scale screening.

## 7. Conclusions and Challenges

In conclusion, iPSCs provide an excellent model to assess the effects of pathogenic factors on ALS-related neurons and glial cells due to their easy access and retention of the genetic background. iPSC technology provides an opportunity to obtain disease-specific and patient-specific iPSCs for human disease modeling, drug screening and cell therapy. It brings new hope for the treatment of ALS.

Although iPSCs technology is a powerful tool, it continuously faces various challenges. (1) Despite the efforts of researchers, the low reprogramming efficiency of iPSCs has limited their future application. Finding a simple and effective method to improve the efficiency of reprogramming is a problem worthy of serious consideration and urgent solution. (2) ALS is a neurodegenerative disease related to age. Since hiPSCs derivatives are more similar to the fetal stage than the adult stage, hiPSCs-derived cells may not capture the correct phenotype of ALS disease. We suggest finding a method to promote aging and applying it to iPSCs-derived cells from ALS patients, including genetic approaches and the upregulation of key drivers of aging regulators. (3) It is time-consuming and expensive to collect patient samples, establish iPSCs culture and induce specific neuron and glial cell differentiation. We propose to prepare cell banks of iPSC derivatives to promote stem cell clinical applications and future commercialization. Of course, attention should be paid to selecting the appropriate cell type, ensuring the quality of cell products and formulating quality standards at various stages of the manufacturing process. (4) While many reports have shown that iPSCs-derived neural cells can replicate the phenotype of ALS, most of these cases come from familial cases related to specific gene mutations. For sALS, it is still challenging to produce cytopathic cells with disease-related phenotypes. We suggest that sALS should be studied using epigenomic analysis and clustering including advanced mathematical and scientific methods and AI techniques to discover and fully identify relevant disease markers. Research conducted to date with aggregated sporadic cases can provide reliable data for further drug discovery and the formulation of treatment strategies.

## Figures and Tables

**Table 1:** Modeling ALS using iPSCs-derived motor neurons, astrocytes and microglia.

Cell Type	ALS Subtypes	Key Findings
Motor neurons (or neurons)	*SOD1* mutation	Neurofilament aggregation; disorders in mitochondria; decreased survival rate
	*C9orf72* mutation	RNA foci formation; accumulation of RAN translation products; stress granule formation; excitotoxicity
	*TARDBP* mutation	TDP-43 protein aggregation; shorter neurites; mitochondrial Ca^2+^ uptake disorder
	*FUS* mutation	FUS protein aggregation; DNA damage; cytoplasmic mislocation
	sporadic ALS	TDP-43 aggregation; higher neurofilament inclusion; mitochondria distribution impairment
Astrocytes	*SOD1* mutation	Impair autophagy
	*C9orf72* mutation	Increased oxidative stress and neurotoxicity
	sporadic ALS	Autophagy-mediated inflammatory cytokine secretion
Microglia	*FUS* mutation	Disrupt intracellular calcium signaling
	sporadic ALS	Influence neurofilament deposition

**Table 2:** Candidate drugs and compounds for ALS developed by iPSCs-disease models.

Name	Functions	Reference
Bosutinib	A Src/c-Abl inhibitor; increases the survival of ALS MNs, increases autophagy, reduces the amount of misfolded SOD1 protein.	[52]
Retigabine (ezogabine)	A Kv7 channel activator; blocks hyperexcitability, improves MN survival.	[80]
ROPI	A dopamine agonist; suppresses neurite retraction and cell death, inhibits oxidative stress, improves mitochondrial function, inhibits TDP-43 and FUS aggregation.	[111]
Compound #56	An inhibitor of SOD1-Derlin-1 interaction; ameliorates ALS pathology in MNs, delays onset and prolongs survival of ALS model mice.	[112]
Mitoxantrone	Contains extended planararomatic moieties; prevents TDP-43, FUS from forming SGs.	[113]
SAHA, RGFP109	Histone deacetylase inhibitors; reduce loss of nuclear FUS, rescue the DNA repair response (combined with arimoclomol).	[114]
Niclosamide	A STAT3 inhibitor; prevents TDP-43 mislocation, degrades TDP-43 aggregates, activates mitophagy, attenuates morphological changes.	[115]

## Data Availability

Not applicable.

## Abbreviations

ALS	amyotrophic lateral sclerosis
CNS	central nervous system
fALS	familial amyotrophic lateral sclerosis
sALS	sporadic amyotrophic lateral sclerosis
FDA	Food and Drug Administration
iPSCs	induced pluripotent stem cells
ESCs	embryonic stem cells
MNs	motor neurons
hiPSCs	human induced pluripotent stem cells
iPSCs-MNs	induced pluripotent stem cells derived motor neurons
ESCs-MNs	embryonic stem cells derived motor neurons
NPCs	neural progenitor cells
iPSCs-NPCs	induced pluripotent stem cells derived neural progenitor cells
NSCs	neural stem cells
iPSCs-NSCs	induced pluripotent stem cells derived neural stem cells
NMJ	neuromuscular junction
SOD1	superoxide dismutase 1
C9orf72	chromosome nine open reading frame 72
TARDBP	TAR DNA binding protein
FUS	fused in sarcoma
NfH	neurofilament heavy chain
NfL	neurofilament light chain
CSF	cerebrospinal fluid
HD-MEAs	high density microelectrode-arrays
DPR	dipeptide repeat
TDP-43	TAR DNA binding protein 43
RNAP II	RNA polymerase II
EAAT2	excitatory amino acid transporter 2
SG	stress granule
G3BP1	GTPase activating protein (SH3 domain) binding protein 1
Il-1α	interleukin 1α
TNF	tumor necrosis factor
C1q	complement component 1, subcomponent q
ROPI	ropinirole
HDAC	histone deacetylase
FTD	frontotemporal dementia
STAT3	signal transducer and activator of transcription 3
TGF-β	transforming growth factor-β

## References

[1] Dafinca R., Scaber J., Ababneh N., Lalic T., Weir G., Christian H., Vowles J., Douglas A.G., Fletcher-Jones A., Browne C. (2016). C9orf72 Hexanucleotide Expansions Are Associated with Altered Endoplasmic Reticulum Calcium Homeostasis and Stress Granule Formation in Induced Pluripotent Stem Cell-Derived Neurons from Patients with Amyotrophic Lateral Sclerosis and Frontotemporal Dementia. Stem Cells.

[2] Pender N., Pinto-Grau M., Hardiman O. (2020). Cognitive and behavioural impairment in amyotrophic lateral sclerosis. Curr. Opin. Neurol..

[3] Feldman E.L., Goutman S.A., Petri S., Mazzini L., Savelieff M.G., Shaw P.J., Sobue G. (2022). Amyotrophic lateral sclerosis. Lancet.

[4] Xu L., Liu T., Liu L., Yao X., Chen L., Fan D., Zhan S., Wang S. (2020). Global variation in prevalence and incidence of amyotrophic lateral sclerosis: A systematic review and meta-analysis. J. Neurol..

[5] Talbott E.O., Malek A., Lacomis D. (2016). The epidemiology of amyotrophic lateral sclerosis. Handb. Clin. Neurol..

[6] Pham J., Keon M., Brennan S., Saksena N. (2020). Connecting RNA-Modifying Similarities of TDP-43, FUS, and SOD1 with MicroRNA Dysregulation Amidst a Renewed Network Perspective of Amyotrophic Lateral Sclerosis Proteinopathy. Int. J. Mol. Sci..

[7] Rosen D.R., Siddique T., Patterson D., Figlewicz D.A., Sapp P., Hentati A., Donaldson D., Goto J., O’Regan J.P., Deng H.X. (1993). Mutations in Cu/Zn superoxide dismutase gene are associated with familial amyotrophic lateral sclerosis. Nature.

[8] Kato S., Kato M., Abe Y., Matsumura T., Nishino T., Aoki M., Itoyama Y., Asayama K., Awaya A., Hirano A. (2005). Redox system expression in the motor neurons in amyotrophic lateral sclerosis (ALS): Immunohistochemical studies on sporadic ALS, superoxide dismutase 1 (SOD1)-mutated familial ALS, and SOD1-mutated ALS animal models. Acta Neuropathol..

[9] Kaur S.J., McKeown S., Rashid S. (2016). Mutant SOD1 mediated pathogenesis of Amyotrophic Lateral Sclerosis. Gene.

[10] Renton A.E., Majounie E., Waite A., Simon-Sanchez J., Rollinson S., Gibbs J.R., Schymick J.C., Laaksovirta H., van Swieten J.C., Myllykangas L. (2011). A hexanucleotide repeat expansion in C9ORF72 is the cause of chromosome 9p21-linked ALS-FTD. Neuron.

[11] Zhang K., Donnelly C.J., Haeusler A.R., Grima J.C., Machamer J.B., Steinwald P., Daley E.L., Miller S.J., Cunningham K.M., Vidensky S. (2015). The C9orf72 repeat expansion disrupts nucleocytoplasmic transport. Nature.

[12] Costa J., Gomes C., de Carvalho M. (2010). Diagnosis, pathogenesis and therapeutic targets in amyotrophic lateral sclerosis. CNS Neurol. Disord. Drug Targets.

[13] Van Harten A.C.M., Phatnani H., Przedborski S. (2021). Non-cell-autonomous pathogenic mechanisms in amyotrophic lateral sclerosis. Trends Neurosci..

[14] Masrori P., Van Damme P. (2020). Amyotrophic lateral sclerosis: A clinical review. Eur. J. Neurol..

[15] Behl T., Kaur G., Sehgal A., Bhardwaj S., Singh S., Buhas C., Judea-Pusta C., Uivarosan D., Munteanu M.A., Bungau S. (2021). Multifaceted Role of Matrix Metalloproteinases in Neurodegenerative Diseases: Pathophysiological and Therapeutic Perspectives. Int. J. Mol. Sci..

[16] Fang X. (2016). Potential role of gut microbiota and tissue barriers in Parkinson’s disease and amyotrophic lateral sclerosis. Int. J. Neurosci..

[17] Zeng Q., Shen J., Chen K., Zhou J., Liao Q., Lu K., Yuan J., Bi F. (2020). The alteration of gut microbiome and metabolism in amyotrophic lateral sclerosis patients. Sci. Rep..

[18] Niccolai E., Di Pilato V., Nannini G., Baldi S., Russo E., Zucchi E., Martinelli I., Menicatti M., Bartolucci G., Mandrioli J. (2021). The Gut Microbiota-Immunity Axis in ALS: A Role in Deciphering Disease Heterogeneity?. Biomedicines.

[19] Nicholson K., Bjornevik K., Abu-Ali G., Chan J., Cortese M., Dedi B., Jeon M., Xavier R., Huttenhower C., Ascherio A. (2021). The human gut microbiota in people with amyotrophic lateral sclerosis. Amyotroph Lateral Scler Front. Degener..

[20] Lenglet T., Camdessanche J. (2017). Amyotrophic lateral sclerosis or not: Keys for the diagnosis. Rev. Neurol..

[21] Huang F., Zhu Y., Hsiao-Nakamoto J., Tang X., Dugas J.C., Moscovitch-Lopatin M., Glass J.D., Brown R.H., Ladha S.S., Lacomis D. (2020). Longitudinal biomarkers in amyotrophic lateral sclerosis. Ann. Clin. Transl. Neurol..

[22] Niedermeyer S., Murn M., Choi P. (2019). Respiratory Failure in Amyotrophic Lateral Sclerosis. Chest.

[23] Rothstein J.D. (2017). Edaravone: A new drug approved for ALS. Cell.

[24] Bensimon G., Lacomblez L., Meininger V. (1994). A controlled trial of riluzole in amyotrophic lateral sclerosis. ALS/Riluzole Study Group. N. Engl. J. Med..

[25] Witzel S., Maier A., Steinbach R., Grosskreutz J., Koch J.C., Sarikidi A., Petri S., Gunther R., Wolf J., Hermann A. (2022). Safety and Effectiveness of Long-term Intravenous Administration of Edaravone for Treatment of Patients with Amyotrophic Lateral Sclerosis. JAMA Neurol..

[26] Aschenbrenner D.S. (2023). New Drug Approved For ALS. Am. J. Nurs..

[27] Miller T.M., Cudkowicz M.E., Genge A., Shaw P.J., Sobue G., Bucelli R.C., Chio A., Van Damme P., Ludolph A.C., Glass J.D. (2022). Trial of Antisense Oligonucleotide Tofersen for SOD1 ALS. N. Engl. J. Med..

[28] Takahashi K., Yamanaka S. (2006). Induction of pluripotent stem cells from mouse embryonic and adult fibroblast cultures by defined factors. Cell.

[29] Takahashi K., Tanabe K., Ohnuki M., Narita M., Ichisaka T., Tomoda K., Yamanaka S. (2007). Induction of pluripotent stem cells from adult human fibroblasts by defined factors. Cell.

[30] Dimos J.T., Rodolfa K.T., Niakan K.K., Weisenthal L.M., Mitsumoto H., Chung W., Croft G.F., Saphier G., Leibel R., Goland R. (2008). Induced pluripotent stem cells generated from patients with ALS can be differentiated into motor neurons. Science.

[31] Ferraiuolo L., Maragakis N. (2021). Mini-Review: Induced pluripotent stem cells and the search for new cell-specific ALS therapeutic targets. Neurosci. Lett..

[32] Ray A., Joshi J.M., Sundaravadivelu P.K., Raina K., Lenka N., Kaveeshwar V., Thummer R.P. (2021). An Overview on Promising Somatic Cell Sources Utilized for the Efficient Generation of Induced Pluripotent Stem Cells. Stem Cell Rev. Rep..

[33] Nakagawa M., Koyanagi M., Tanabe K., Takahashi K., Ichisaka T., Aoi T., Okita K., Mochiduki Y., Takizawa N., Yamanaka S. (2008). Generation of induced pluripotent stem cells without Myc from mouse and human fibroblasts. Nat. Biotechnol..

[34] Zhou W., Freed C. (2009). Adenoviral gene delivery can reprogram human fibroblasts to induced pluripotent stem cells. Stem Cells.

[35] Haase A., Olmer R., Schwanke K., Wunderlich S., Merkert S., Hess C., Zweigerdt R., Gruh I., Meyer J., Wagner S. (2009). Generation of induced pluripotent stem cells from human cord blood. Cell Stem Cell.

[36] Kim D., Kim C.H., Moon J.I., Chung Y.G., Chang M.Y., Han B.S., Ko S., Yang E., Cha K.Y., Lanza R. (2009). Generation of human induced pluripotent stem cells by direct delivery of reprogramming proteins. Cell Stem Cell.

[37] Yakubov E., Rechavi G., Rozenblatt S., Givol D. (2010). Reprogramming of human fibroblasts to pluripotent stem cells using mRNA of four transcription factors. Biochem. Biophys. Res. Commun..

[38] Fusaki N., Ban H., Nishiyama A., Saeki K., Hasegawa M. (2009). Efficient induction of transgene-free human pluripotent stem cells using a vector based on Sendai virus, an RNA virus that does not integrate into the host genome. Proc. Jpn. Acad. Ser. B Phys. Biol. Sci..

[39] Zhang Y., Zhu J., Dai Y., Wang L., Liu R., Guo X. (2022). Generation of an induced pluripotent stem cell line (ZZUi034-A) from a 65 year old Chinese female donor with sendai virus reprogramming protocol. Stem Cell Res..

[40] Hou P., Li Y., Zhang X., Liu C., Guan J., Li H., Zhao T., Ye J., Yang W., Liu K. (2013). Pluripotent stem cells induced from mouse somatic cells by small-molecule compounds. Science.

[41] Chen K., Long Q., Wang T., Zhao D., Zhou Y., Qi J., Wu Y., Li S., Chen C., Zeng X. (2016). Gadd45a is a heterochromatin relaxer that enhances iPS cell generation. EMBO Rep..

[42] Haraguchi D., Nakamura T. (2022). Pramef12 enhances reprogramming into naive iPS cells. Biochem. Biophys. Rep..

[43] Lee S., Huang E. (2017). Modeling ALS and FTD with iPSC-derived neurons. Brain Res..

[44] Lamas N.J., Roybon L. (2021). Harnessing the Potential of Human Pluripotent Stem Cell-Derived Motor Neurons for Drug Discovery in Amyotrophic Lateral Sclerosis: From the Clinic to the Laboratory and Back to the Patient. Front. Drug Discov..

[45] Castillo Bautista C.M., Sterneckert J. (2022). Progress and challenges in directing the differentiation of human iPSCs into spinal motor neurons. Front. Cell Dev. Biol..

[46] Son E.Y., Ichida J.K., Wainger B.J., Toma J.S., Rafuse V.F., Woolf C.J., Eggan K. (2011). Conversion of mouse and human fibroblasts into functional spinal motor neurons. Cell Stem Cell.

[47] Ronchi S., Buccino A.P., Prack G., Kumar S.S., Schroter M., Fiscella M., Hierlemann A. (2021). Electrophysiological Phenotype Characterization of Human iPSC-Derived Neuronal Cell Lines by Means of High-Density Microelectrode Arrays. Adv. Biol..

[48] Toma J.S., Shettar B.C., Chipman P.H., Pinto D.M., Borowska J.P., Ichida J.K., Fawcett J.P., Zhang Y., Eggan K., Rafuse V.F. (2015). Motoneurons derived from induced pluripotent stem cells develop mature phenotypes typical of endogenous spinal motoneurons. J. Neurosci..

[49] Azadmanesh J., Borgstahl G. (2018). A Review of the Catalytic Mechanism of Human Manganese Superoxide Dismutase. Antioxidants.

[50] Chen H., Qian K., Du Z., Cao J., Petersen A., Liu H., Blackbourn L.W.t., Huang C.L., Errigo A., Yin Y. (2014). Modeling ALS with iPSCs reveals that mutant SOD1 misregulates neurofilament balance in motor neurons. Cell Stem Cell.

[51] Kiskinis E., Sandoe J., Williams L.A., Boulting G.L., Moccia R., Wainger B.J., Han S., Peng T., Thams S., Mikkilineni S. (2014). Pathways disrupted in human ALS motor neurons identified through genetic correction of mutant SOD1. Cell Stem Cell.

[52] Imamura K., Izumi Y., Watanabe A., Tsukita K., Woltjen K., Yamamoto T., Hotta A., Kondo T., Kitaoka S., Ohta A. (2017). The Src/c-Abl pathway is a potential therapeutic target in amyotrophic lateral sclerosis. Sci. Transl. Med..

[53] Zou Z.Y., Zhou Z.R., Che C.H., Liu C.Y., He R.L., Huang H.P. (2017). Genetic epidemiology of amyotrophic lateral sclerosis: A systematic review and meta-analysis. J. Neurol Neurosurg. Psychiatry.

[54] Donnelly C.J., Zhang P.W., Pham J.T., Haeusler A.R., Mistry N.A., Vidensky S., Daley E.L., Poth E.M., Hoover B., Fines D.M. (2013). RNA toxicity from the ALS/FTD C9ORF72 expansion is mitigated by antisense intervention. Neuron.

[55] Almeida S., Gascon E., Tran H., Chou H.J., Gendron T.F., Degroot S., Tapper A.R., Sellier C., Charlet-Berguerand N., Karydas A. (2013). Modeling key pathological features of frontotemporal dementia with C9ORF72 repeat expansion in iPSC-derived human neurons. Acta Neuropathol..

[56] Westergard T., McAvoy K., Russell K., Wen X., Pang Y., Morris B., Pasinelli P., Trotti D., Haeusler A. (2019). Repeat-associated non-AUG translation in C9orf72-ALS/FTD is driven by neuronal excitation and stress. EMBO Mol. Med..

[57] Sakae N., Bieniek K.F., Zhang Y.J., Ross K., Gendron T.F., Murray M.E., Rademakers R., Petrucelli L., Dickson D.W. (2018). Poly-GR dipeptide repeat polymers correlate with neurodegeneration and Clinicopathological subtypes in C9ORF72-related brain disease. Acta Neuropathol. Commun..

[58] Lopez-Gonzalez R., Lu Y., Gendron T.F., Karydas A., Tran H., Yang D., Petrucelli L., Miller B.L., Almeida S., Gao F.B. (2016). Poly(GR) in C9ORF72-Related ALS/FTD Compromises Mitochondrial Function and Increases Oxidative Stress and DNA Damage in iPSC-Derived Motor Neurons. Neuron.

[59] Freibaum B.D., Lu Y., Lopez-Gonzalez R., Kim N.C., Almeida S., Lee K.H., Badders N., Valentine M., Miller B.L., Wong P.C. (2015). GGGGCC repeat expansion in C9orf72 compromises nucleocytoplasmic transport. Nature.

[60] Dafinca R., Barbagallo P., Farrimond L., Candalija A., Scaber J., Ababneh N.A., Sathyaprakash C., Vowles J., Cowley S.A., Talbot K. (2020). Impairment of Mitochondrial Calcium Buffering Links Mutations in C9ORF72 and TARDBP in iPS-Derived Motor Neurons from Patients with ALS/FTD. Stem Cell Rep..

[61] Gomez-Suaga P., Morotz G.M., Markovinovic A., Martin-Guerrero S.M., Preza E., Arias N., Mayl K., Aabdien A., Gesheva V., Nishimura A. (2022). Disruption of ER-mitochondria tethering and signalling in C9orf72-associated amyotrophic lateral sclerosis and frontotemporal dementia. Aging Cell.

[62] Neuro L.C., Li J., Lim R.G., Kaye J.A., Dardov V., Coyne A.N., Wu J., Milani P., Cheng A., Thompson T.G. (2021). An integrated multi-omic analysis of iPSC-derived motor neurons from C9ORF72 ALS patients. iScience.

[63] Bilican B., Serio A., Barmada S.J., Nishimura A.L., Sullivan G.J., Carrasco M., Phatnani H.P., Puddifoot C.A., Story D., Fletcher J. (2012). Mutant induced pluripotent stem cell lines recapitulate aspects of TDP-43 proteinopathies and reveal cell-specific vulnerability. Proc. Natl. Acad. Sci. USA.

[64] Egawa N., Kitaoka S., Tsukita K., Naitoh M., Takahashi K., Yamamoto T., Adachi F., Kondo T., Okita K., Asaka I. (2012). Drug screening for ALS using patient-specific induced pluripotent stem cells. Sci. Transl. Med..

[65] Kreiter N., Pal A., Lojewski X., Corcia P., Naujock M., Reinhardt P., Sterneckert J., Petri S., Wegner F., Storch A. (2018). Age-dependent neurodegeneration and organelle transport deficiencies in mutant TDP43 patient-derived neurons are independent of TDP43 aggregation. Neurobiol. Dis..

[66] Yu C.H., Davidson S., Harapas C.R., Hilton J.B., Mlodzianoski M.J., Laohamonthonkul P., Louis C., Low R.R.J., Moecking J., De Nardo D. (2020). TDP-43 Triggers Mitochondrial DNA Release via mPTP to Activate cGAS/STING in ALS. Cell.

[67] Higelin J., Demestre M., Putz S., Delling J.P., Jacob C., Lutz A.K., Bausinger J., Huber A.K., Klingenstein M., Barbi G. (2016). FUS Mislocalization and Vulnerability to DNA Damage in ALS Patients Derived hiPSCs and Aging Motoneurons. Front. Cell Neurosci..

[68] Hofweber M., Hutten S., Bourgeois B., Spreitzer E., Niedner-Boblenz A., Schifferer M., Ruepp M.D., Simons M., Niessing D., Madl T. (2018). Phase Separation of FUS Is Suppressed by Its Nuclear Import Receptor and Arginine Methylation. Cell.

[69] Reber S., Jutzi D., Lindsay H., Devoy A., Mechtersheimer J., Levone B.R., Domanski M., Bentmann E., Dormann D., Muhlemann O. (2021). The phase separation-dependent FUS interactome reveals nuclear and cytoplasmic function of liquid-liquid phase separation. Nucleic Acids Res..

[70] Liu X., Chen J., Liu W., Li X., Chen Q., Liu T., Gao S., Deng M. (2015). The fused in sarcoma protein forms cytoplasmic aggregates in motor neurons derived from integration-free induced pluripotent stem cells generated from a patient with familial amyotrophic lateral sclerosis carrying the FUS-P525L mutation. Neurogenetics.

[71] Marrone L., Poser I., Casci I., Japtok J., Reinhardt P., Janosch A., Andree C., Lee H.O., Moebius C., Koerner E. (2018). Isogenic FUS-eGFP iPSC Reporter Lines Enable Quantification of FUS Stress Granule Pathology that Is Rescued by Drugs Inducing Autophagy. Stem Cell Rep..

[72] Guo W., Naujock M., Fumagalli L., Vandoorne T., Baatsen P., Boon R., Ordovas L., Patel A., Welters M., Vanwelden T. (2017). HDAC6 inhibition reverses axonal transport defects in motor neurons derived from FUS-ALS patients. Nat. Commun..

[73] Picchiarelli G., Demestre M., Zuko A., Been M., Higelin J., Dieterle S., Goy M.A., Mallik M., Sellier C., Scekic-Zahirovic J. (2019). FUS-mediated regulation of acetylcholine receptor transcription at neuromuscular junctions is compromised in amyotrophic lateral sclerosis. Nat. Neurosci..

[74] Tsai Y.L., Mu Y., Manley J. (2022). Nuclear RNA transcript levels modulate nucleocytoplasmic distribution of ALS/FTD-associated protein FUS. Sci. Rep..

[75] Gubert F., Vasques J.F., Cozendey T.D., Domizi P., Toledo M.F., Kasai-Brunswick T.H., Loureiro M.P.S., Lima J.M.B., Gress C.H., Cabello G.M.K. (2019). Generation of four patient-specific pluripotent induced stem cell lines from two Brazilian patients with amyotrophic lateral sclerosis and two healthy subjects. Stem Cell Res..

[76] Burkhardt M.F., Martinez F.J., Wright S., Ramos C., Volfson D., Mason M., Garnes J., Dang V., Lievers J., Shoukat-Mumtaz U. (2013). A cellular model for sporadic ALS using patient-derived induced pluripotent stem cells. Mol. Cell Neurosci..

[77] Alves C.J., Dariolli R., Jorge F.M., Monteiro M.R., Maximino J.R., Martins R.S., Strauss B.E., Krieger J.E., Callegaro D., Chadi G. (2015). Gene expression profiling for human iPS-derived motor neurons from sporadic ALS patients reveals a strong association between mitochondrial functions and neurodegeneration. Front. Cell Neurosci..

[78] Sun X., Song J., Huang H., Chen H., Qian K. (2018). Modeling hallmark pathology using motor neurons derived from the family and sporadic amyotrophic lateral sclerosis patient-specific iPS cells. Stem Cell Res. Ther..

[79] Baxi E.G., Thompson T., Li J., Kaye J.A., Lim R.G., Wu J., Ramamoorthy D., Lima L., Vaibhav V., Matlock A. (2022). Answer ALS, a large-scale resource for sporadic and familial ALS combining clinical and multi-omics data from induced pluripotent cell lines. Nat. Neurosci..

[80] Wainger B.J., Kiskinis E., Mellin C., Wiskow O., Han S.S., Sandoe J., Perez N.P., Williams L.A., Lee S., Boulting G. (2014). Intrinsic membrane hyperexcitability of amyotrophic lateral sclerosis patient-derived motor neurons. Cell Rep..

[81] Bristol L.A., Rothstein J. (1996). Glutamate transporter gene expression in amyotrophic lateral sclerosis motor cortex. Ann. Neurol..

[82] Tyzack G., Lakatos A., Patani R. (2016). Human Stem Cell-Derived Astrocytes: Specification and Relevance for Neurological Disorders. Curr. Stem Cell Rep..

[83] Muffat J., Li Y., Yuan B., Mitalipova M., Omer A., Corcoran S., Bakiasi G., Tsai L.H., Aubourg P., Ransohoff R.M. (2016). Efficient derivation of microglia-like cells from human pluripotent stem cells. Nat. Med..

[84] Madill M., McDonagh K., Ma J., Vajda A., McLoughlin P., O’Brien T., Hardiman O., Shen S. (2017). Amyotrophic lateral sclerosis patient iPSC-derived astrocytes impair autophagy via non-cell autonomous mechanisms. Mol. Brain.

[85] Birger A., Ben-Dor I., Ottolenghi M., Turetsky T., Gil Y., Sweetat S., Perez L., Belzer V., Casden N., Steiner D. (2019). Human iPSC-derived astrocytes from ALS patients with mutated C9ORF72 show increased oxidative stress and neurotoxicity. EBioMedicine.

[86] Zhao C., Devlin A.C., Chouhan A.K., Selvaraj B.T., Stavrou M., Burr K., Brivio V., He X., Mehta A.R., Story D. (2020). Mutant C9orf72 human iPSC-derived astrocytes cause non-cell autonomous motor neuron pathophysiology. Glia.

[87] Rajpurohit C.S., Kumar V., Cheffer A., Oliveira D., Ulrich H., Okamoto O.K., Zatz M., Ansari U.A., Khanna V.K., Pant A.B. (2020). Mechanistic Insights of Astrocyte-Mediated Hyperactive Autophagy and Loss of Motor Neuron Function in SOD1(L39R) Linked Amyotrophic Lateral Sclerosis. Mol. Neurobiol..

[88] Feng B., Amponsah A.E., Guo R., Liu X., Zhang J., Du X., Zhou Z., He J., Ma J., Cui H. (2022). Autophagy-Mediated Inflammatory Cytokine Secretion in Sporadic ALS Patient iPSC-Derived Astrocytes. Oxid. Med. Cell Longev..

[89] Allison R.L., Adelman J.W., Abrudan J., Urrutia R.A., Zimmermann M.T., Mathison A.J., Ebert A.D. (2022). Microglia Influence Neurofilament Deposition in ALS iPSC-Derived Motor Neurons. Genes.

[90] Kerk S.Y., Bai Y., Smith J., Lalgudi P., Hunt C., Kuno J., Nuara J., Yang T., Lanza K., Chan N. (2022). Homozygous ALS-linked FUS P525L mutations cell-autonomously perturb transcriptome profile and chemoreceptor signaling in human iPSC microglia. Stem Cell Rep..

[91] Eitan C., Siany A., Barkan E., Olender T., van Eijk K.R., Moisse M., Farhan S.M.K., Danino Y.M., Yanowski E., Marmor-Kollet H. (2022). Whole-genome sequencing reveals that variants in the Interleukin 18 Receptor Accessory Protein 3’UTR protect against ALS. Nat. Neurosci..

[92] Barres B.A. (2008). The mystery and magic of glia: A perspective on their roles in health and disease. Neuron.

[93] Liddelow S.A., Guttenplan K.A., Clarke L.E., Bennett F.C., Bohlen C.J., Schirmer L., Bennett M.L., Munch A.E., Chung W.S., Peterson T.C. (2017). Neurotoxic reactive astrocytes are induced by activated microglia. Nature.

[94] Jarrige M., Frank E., Herardot E., Martineau S., Darle A., Benabides M., Domingues S., Chose O., Habeler W., Lorant J. (2021). The Future of Regenerative Medicine: Cell Therapy Using Pluripotent Stem Cells and Acellular Therapies Based on Extracellular Vesicles. Cells.

[95] Clement A.M., Nguyen M.D., Roberts E.A., Garcia M.L., Boillee S., Rule M., McMahon A.P., Doucette W., Siwek D., Ferrante R.J. (2003). Wild-type nonneuronal cells extend survival of SOD1 mutant motor neurons in ALS mice. Science.

[96] Ghasemi M., Keyhanian K., Douthwright C. (2021). Glial Cell Dysfunction in C9orf72-Related Amyotrophic Lateral Sclerosis and Frontotemporal Dementia. Cells.

[97] Sareen D., Gowing G., Sahabian A., Staggenborg K., Paradis R., Avalos P., Latter J., Ornelas L., Garcia L., Svendsen C.N. (2014). Human induced pluripotent stem cells are a novel source of neural progenitor cells (iNPCs) that migrate and integrate in the rodent spinal cord. J. Comp. Neurol..

[98] Kondo T., Funayama M., Tsukita K., Hotta A., Yasuda A., Nori S., Kaneko S., Nakamura M., Takahashi R., Okano H. (2014). Focal transplantation of human iPSC-derived glial-rich neural progenitors improves lifespan of ALS mice. Stem Cell Rep..

[99] Popescu I.R., Nicaise C., Liu S., Bisch G., Knippenberg S., Daubie V., Bohl D., Pochet R. (2013). Neural progenitors derived from human induced pluripotent stem cells survive and differentiate upon transplantation into a rat model of amyotrophic lateral sclerosis. Stem Cells Transl. Med..

[100] Nizzardo M., Simone C., Rizzo F., Ruggieri M., Salani S., Riboldi G., Faravelli I., Zanetta C., Bresolin N., Comi G.P. (2014). Minimally invasive transplantation of iPSC-derived ALDHhiSSCloVLA4+ neural stem cells effectively improves the phenotype of an amyotrophic lateral sclerosis model. Hum. Mol. Genet..

[101] Nizzardo M., Bucchia M., Ramirez A., Trombetta E., Bresolin N., Comi G.P., Corti S. (2016). iPSC-derived LewisX+CXCR4+beta1-integrin+ neural stem cells improve the amyotrophic lateral sclerosis phenotype by preserving motor neurons and muscle innervation in human and rodent models. Hum. Mol. Genet..

[102] Rosati J., Ferrari D., Altieri F., Tardivo S., Ricciolini C., Fusilli C., Zalfa C., Profico D.C., Pinos F., Bernardini L. (2018). Establishment of stable iPS-derived human neural stem cell lines suitable for cell therapies. Cell Death Dis..

[103] Forostyak S., Forostyak O., Kwok J.C.F., Romanyuk N., Rehorova M., Kriska J., Dayanithi G., Raha-Chowdhury R., Jendelova P., Anderova M. (2020). Transplantation of Neural Precursors Derived from Induced Pluripotent Cells Preserve Perineuronal Nets and Stimulate Neural Plasticity in ALS Rats. Int. J. Mol. Sci..

[104] Liu B., Li M., Zhang L., Chen Z., Lu P. (2022). Motor neuron replacement therapy for amyotrophic lateral sclerosis. Neural Regen. Res..

[105] Sun Y., Feng L., Liang L., Stacey G.N., Wang C., Wang Y., Hu B. (2021). Neuronal cell-based medicines from pluripotent stem cells: Development, production, and preclinical assessment. Stem Cells Transl. Med..

[106] Ting H.C., Su H.L., Chen M.F., Harn H.J., Lin S.Z., Chiou T.W., Chang C.Y. (2022). Robust Generation of Ready-to-Use Cryopreserved Motor Neurons from Human Pluripotent Stem Cells for Disease Modeling. Int. J. Mol. Sci..

[107] Hayashi Y., Ohnuma K., Furue M. (2019). Pluripotent Stem Cell Heterogeneity. Adv. Exp. Med. Biol..

[108] Bar S., Benvenisty N. (2019). Epigenetic aberrations in human pluripotent stem cells. EMBO J..

[109] Giacomelli E., Vahsen B.F., Calder E.L., Xu Y., Scaber J., Gray E., Dafinca R., Talbot K., Studer L. (2022). Human stem cell models of neurodegeneration: From basic science of amyotrophic lateral sclerosis to clinical translation. Cell Stem Cell.

[110] Ohnuki M., Takahashi K. (2015). Present and future challenges of induced pluripotent stem cells. Philos. Trans. R. Soc. Lond. B Biol. Sci..

[111] Okano H., Yasuda D., Fujimori K., Morimoto S., Takahashi S. (2020). Ropinirole, a New ALS Drug Candidate Developed Using iPSCs. Trends Pharmacol. Sci..

[112] Tsuburaya N., Homma K., Higuchi T., Balia A., Yamakoshi H., Shibata N., Nakamura S., Nakagawa H., Ikeda S.I., Umezawa N. (2018). A small-molecule inhibitor of SOD1-Derlin-1 interaction ameliorates pathology in an ALS mouse model. Nat. Commun..

[113] Fang M.Y., Markmiller S., Vu A.Q., Javaherian A., Dowdle W.E., Jolivet P., Bushway P.J., Castello N.A., Baral A., Chan M.Y. (2019). Small-Molecule Modulation of TDP-43 Recruitment to Stress Granules Prevents Persistent TDP-43 Accumulation in ALS/FTD. Neuron.

[114] Kuta R., Larochelle N., Fernandez M., Pal A., Minotti S., Tibshirani M., St Louis K., Gentil B.J., Nalbantoglu J.N., Hermann A. (2020). Depending on the stress, histone deacetylase inhibitors act as heat shock protein co-inducers in motor neurons and potentiate arimoclomol, exerting neuroprotection through multiple mechanisms in ALS models. Cell Stress Chaperones.

[115] Kato Y., Sakamoto K. (2021). Niclosamide affects intracellular TDP-43 distribution in motor neurons, activates mitophagy, and attenuates morphological changes under stress. J. Biosci. Bioeng..

[116] Imamura K., Izumi Y., Banno H., Uozumi R., Morita S., Egawa N., Ayaki T., Nagai M., Nishiyama K., Watanabe Y. (2019). Induced pluripotent stem cell-based Drug Repurposing for Amyotrophic lateral sclerosis Medicine (iDReAM) study: Protocol for a phase I dose escalation study of bosutinib for amyotrophic lateral sclerosis patients. BMJ Open.

[117] Imamura K., Izumi Y., Nagai M., Nishiyama K., Watanabe Y., Hanajima R., Egawa N., Ayaki T., Oki R., Fujita K. (2022). Safety and tolerability of bosutinib in patients with amyotrophic lateral sclerosis (iDReAM study): A multicentre, open-label, dose-escalation phase 1 trial. EClinicalMedicine.

[118] Fujimori K., Ishikawa M., Otomo A., Atsuta N., Nakamura R., Akiyama T., Hadano S., Aoki M., Saya H., Sobue G. (2018). Modeling sporadic ALS in iPSC-derived motor neurons identifies a potential therapeutic agent. Nat. Med..

[119] Morimoto S., Takahashi S., Fukushima K., Saya H., Suzuki N., Aoki M., Okano H., Nakahara J. (2019). Ropinirole hydrochloride remedy for amyotrophic lateral sclerosis—Protocol for a randomized, double-blind, placebo-controlled, single-center, and open-label continuation phase I/IIa clinical trial (ROPALS trial). Regen. Ther..

[120] Sleutjes B., Stikvoort Garcia D.J.L., Kovalchuk M.O., Heuberger J., Groeneveld G.J., Franssen H., van den Berg L.H. (2022). Acute retigabine-induced effects on myelinated motor axons in amyotrophic lateral sclerosis. Pharmacol. Res. Perspect..

[121] Wainger B.J., Macklin E.A., Vucic S., McIlduff C.E., Paganoni S., Maragakis N.J., Bedlack R., Goyal N.A., Rutkove S.B., Lange D.J. (2021). Effect of Ezogabine on Cortical and Spinal Motor Neuron Excitability in Amyotrophic Lateral Sclerosis: A Randomized Clinical Trial. JAMA Neurol..

[122] Choi S.Y., Lee J.H., Chung A.Y., Jo Y., Shin J.H., Park H.C., Kim H., Lopez-Gonzalez R., Ryu J.R., Sun W. (2020). Prevention of mitochondrial impairment by inhibition of protein phosphatase 1 activity in amyotrophic lateral sclerosis. Cell Death Dis..

[123] De Jongh R., Spijkers X.M., Pasteuning-Vuhman S., Vulto P., Pasterkamp R.J. (2021). Neuromuscular junction-on-a-chip: ALS disease modeling and read-out development in microfluidic devices. J. Neurochem..

[124] Namboori S.C., Thomas P., Ames R., Hawkins S., Garrett L.O., Willis C.R.G., Rosa A., Stanton L.W., Bhinge A. (2021). Single-cell transcriptomics identifies master regulators of neurodegeneration in SOD1 ALS iPSC-derived motor neurons. Stem Cell Rep..

[125] Deneault E., Chaineau M., Nicouleau M., Castellanos Montiel M.J., Franco Flores A.K., Haghi G., Chen C.X., Abdian N., Shlaifer I., Beitel L.K. (2022). A streamlined CRISPR workflow to introduce mutations and generate isogenic iPSCs for modeling amyotrophic lateral sclerosis. Methods.

[126] Imamura K., Yada Y., Izumi Y., Morita M., Kawata A., Arisato T., Nagahashi A., Enami T., Tsukita K., Kawakami H. (2021). Prediction Model of Amyotrophic Lateral Sclerosis by Deep Learning with Patient Induced Pluripotent Stem Cells. Ann. Neurol..

